# Effect of Pore Connectivity on the Behavior of Fluids Confined in Sub-Nanometer Pores: Ethane and CO_2_ Confined in ZSM-22

**DOI:** 10.3390/membranes11020113

**Published:** 2021-02-05

**Authors:** Mohammed Musthafa Kummali, David Cole, Siddharth Gautam

**Affiliations:** 1 Department of Physics, The New College (Autonomous), 147 Peters Road, Chennai 600014, India; musthafakummali@thenewcollege.edu.in; 2 School of Earth Sciences, The Ohio State University, 125 South Oval Mall, Columbus, OH 43210, USA; cole.618@osu.edu

**Keywords:** CO_2_, ethane, zeolites, MD simulations, pore connectivity

## Abstract

The behavior of fluids under nano-confinement varies from that in bulk due to an interplay of several factors including pore connectivity. In this work, we use molecular dynamics simulations to study the behavior of two fluids—ethane and CO_2_ confined in ZSM-22, a zeolite with channel-like pores of diameter 0.55 nm isolated from each other. By comparing the behavior of the two fluids in ZSM-22 with that reported earlier in ZSM-5, a zeolite with pores of similar shape and size connected to each other via sinusoidal pores running perpendicular to them, we reveal the important role of pore connectivity. Further, by artificially imposing pore connectivity in ZSM-22 via inserting a 2-dimensional slab-like inter-crystalline space of thickness 0.5 nm, we also studied the effect of the dimensionality and geometry of pore connectivity. While the translational motion of both ethane and CO_2_ in ZSM-22 is suppressed as a result of connecting the pores by perpendicular quasi-one-dimensional pores of similar dimensions, the effect of connecting the pores by inserting the inter-crystalline space is different on the translational motion of the two fluids. For ethane, pores connected via inter-crystalline space facilitate translational motion but suppress rotational motion, whereas in the case of CO_2_, both types of motion are suppressed by pore connection due to the strong interaction of CO_2_ with the surface of the substrate.

## 1. Introduction

Zeolites are microporous materials which are commercially used as adsorbents in size and shape selective separation of molecular species and as catalysts in the petrochemical industry [1]. Due to their well-ordered pore structure, guest molecules adsorbed in zeolites exhibit behavior that differs considerably from the behavior of these molecules in their bulk systems [2]. This deviation of the behavior of guest molecules from the bulk is significantly enhanced for pores with smaller dimensions. This happens, for example, in zeolites like ZSM-5, which has a network of interconnected channel-like pores of diameter 0.55 nm (0.56 × 0.53 nm^2^ in the *a*–*b* plane running along *c* axis) [3]. The strict geometrical restriction imposed by smaller pores of this dimension results in strong confinement of the guest molecules and leads to anomalous diffusion. This anomalous diffusion, which is mostly sub-diffusive, can in some severe cases even lead to single-file diffusion [4]. In addition to the geometrical restriction imposed by the nano-porous substrate, interactions between the guest molecule and the substrate can also put severe limitations on the diffusion of guest molecules. In particular, strong electrostatic interactions between the guest and the substrate can immobilize the guest molecules [5,6]. The presence of electrostatic interactions is responsible for the difference in the behavior of small hydrocarbons that are apolar and CO_2_ which has a quadrupole moment. This difference makes CO_2_ preferentially adsorb on a silica surface by displacing apolar hydrocarbons [7,8,9,10]. This strong preferential adsorption of CO_2_ due to its quadrupole moment also facilitates the separation of CO_2_ from a mixture of hydrocarbons [11,12].

The dynamics of guest molecules through nano-pores of a zeolite membrane is an important aspect of membrane technology. While the geometry and dimensions of the confining pores have a strong effect on the behavior of the guest molecules, the character of the inter-connectivity can also play an important role [13]. Although the effects of pore size and shape on the behavior of guest molecules have been extensively studied, the effects of pore connectivity remain relatively less explored. For this purpose, zeolites ZSM-5 and ZSM-22 provide a very good opportunity because both have a network of similar channel-like pores of ~0.55 nm diameter oriented parallel to the crystallographic axis *b* in ZSM-5 and *c* in ZSM-22, but with interconnecting pores in the *a–c* plane in a sinusoidal arrangement in the former versus isolated channels in ZSM-22. A comparison of the behavior of guest molecules in ZSM-5 and ZSM-22 can therefore reveal important insights on the role of pore connectivity in the behavior of guest molecules confined in sub-nanometer pores. Although the effects of pore connectivity involve a variety of other factors including the degrees of connectivity, pore volume, and surface areas, the simple comparison mentioned above can be a first step toward understanding the complex interplay between these factors.

To understand the role of electrostatic interactions and pore connectivity on the structure and dynamics of guest molecules confined in sub-nanometer pores of zeolitic materials, we report here MD simulation studies on ethane or CO_2_ confined in ZSM-22 pores. In this work, we study the different ways that the inter-connectivity of pores in a zeolite membrane may affect the structure and dynamics of typical guest molecules, thereby revealing important insights on this aspect of membrane technology. Comparative studies of the behavior of ethane and CO_2_ to understand the role of the quadrupole moment of CO_2_ have also been reported earlier [14,15]. The results obtained here for ZSM-22 are compared with those reported for ZSM-5 earlier [14] to understand the role of pore connectivity. Further, in some simulations reported here, the pores of ZSM-22 are connected artificially by inserting empty space connecting the pores. These simulations show that the quadrupole moment of CO_2_ and the pore connectivity of the substrate play an important role in determining the behavior of the confined species. We describe all the simulations carried out and reported here in Section 2, after which the salient results from them are listed in Section 3. In Section 4, we discuss the implications of the results and how they reveal the role of pore connectivity. Finally, we list the salient conclusions that can be made from the simulations in Section 5.

## 2. Materials and Simulation Details

**Figure 1:**
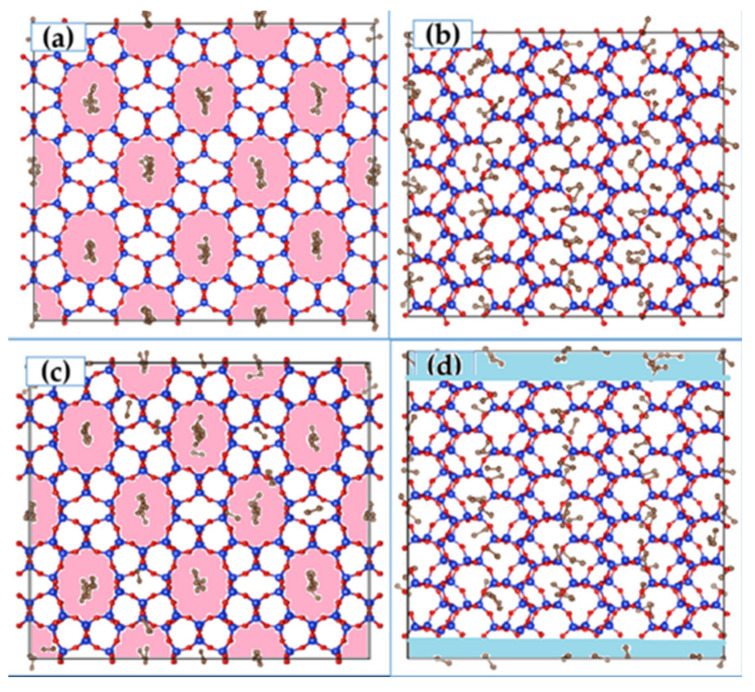
Snapshots from the simulation of ethane in ZSM-22 in X–Y (**a**,**c**) and X–Z (**b**,**d**) planes. (**a**,**b**) show the ZSM-22 simulation cell without any empty space inserted, while the simulation cell in (**c**,**d**) has empty space inserted in the Z-direction. Elliptical channels running in the Z-direction can be seen in the X–Y plane (**a**,**c**) (regions colored in light pink) whereas the empty space inserted in the Z-direction can be seen in (**d**) (slab-like regions highlighted in light blue). Blue, red, and gray spheres, respectively, represent Si, O, and CH_3_ pseudo-atoms.

The crystallographic unit cell of ZSM-22 has 24 Si and 48 O atoms. ZSM-22 has the Theta-1 structure type and contains one-dimensional ten membered-ring pores (running along the Cartesian Z-direction) with channel diameters 4.5 × 5.5 Å^2^. ZSM-22 has an orthorhombic crystal structure with lattice constant *a* = 13.86, *b* = 17.41, and *c* = 5.04 Å [16,17]. In this work, a unit cell of ZSM-22 was replicated 3 × 2 × 6 times using the visualization software VESTA [18] to get the starting simulation cell. Ethane or CO_2_ molecules were loaded into the pores of ZSM-22 in this cell using grand canonical Monte Carlo (GCMC) simulations. MD simulations were performed for three different loadings of ethane and CO_2_ molecules viz. 24, 52, and 72 molecules with a minimum of two, four, and six molecules in each pore. Periodic boundary conditions were applied in all directions during the simulations. Further, an additional simulation was performed at 52 molecular loading of CO_2_ by excluding the Coulombic interactions. Thus, a total of seven simulations were done, three for ethane and four for CO_2_. In addition, to investigate the effects of pore connectivity, an empty space of 2.5 Å was inserted on the top and bottom of the ZSM-22 cell in the Z-direction, thereby exposing the pores to each other. With periodic boundary conditions applied, this renders the pores of ZSM-22 connected to each other via a 2-dimensional inter-crystalline space of thickness 5 Å. Simulations with a loading of 72 ethane or CO_2_ molecules were then carried out in this modified ZSM-22 simulation cell with inter-crystalline space inserted. The highest loading of 72 molecules was considered for this simulation to increase the probability of the guest molecules migrating across the channels. Simulation snapshots showing the unmodified and modified cells are shown in Figure 1.

The interactions between the adsorbate molecules and those between the zeolites and the adsorbates were modeled in terms of the Lennard–Jones (LJ) potential in addition to the Coulombic interactions between entities with partial electrostatic charges. In general, the intermolecular force field took the following form:

$$U_{ij}=4\varepsilon_{ij}\left[{\left(\frac{\sigma_{ij}}{r_{ij}}\right)}^{12}-{\left(\frac{\sigma_{ij}}{r_{ij}}\right)}^{6}\right]+\frac{q_{i}q_{j}}{4\pi \epsilon_{0}r_{ij}}$$

where *ε_ij_* is the depth of the potential well, *σ_ij_* is the distance at which the intermolecular potential between the atoms *i* and *j* becomes zero, the van der Waals radius, and *r_ij_* is the distance between atoms *i*, and *j*, *q_i_*, and *q_j_* are the charges of the *i* and *j* atoms. TraPPE-UA [19] force field was used to model the interaction between the guest molecules and united atom formalism was used to model ethane molecules with two CH_3_ pseudo-atoms connected to each other. Both CO_2_ as well as ethane were considered as rigid molecules with fixed bond lengths, *l*(C-O_C_) = 0.116 nm (O_c_ is used to denote the oxygen atom belonging to a CO_2_ molecule) and *l*(CH_3_-CH_3_) = 0.154 nm. ZSM-22 was modeled using the CLAYFF force field [20]. All ZSM-22 atoms were kept fixed during all simulations. Values of the Lennard–Jones parameters corresponding to CO_2_ and ethane molecules have been summarized in Table 1 and Table 2, respectively. The partial charges on the atoms of ZSM-22 and CO_2_ were taken as *q_Si_* = +2.1*e*, *q_O_* = −1.05*e*, *q_c_* = +0.7*e*, *q_Oc_* = −0.35*e*. The force-field parameters used in this work have been used earlier for simulating the adsorption, structure, and dynamics of ethane and CO_2_ in silicalite—an all silica analogue of ZSM-5 [14,15]. The adsorption isotherms of both ethane and CO_2_ in silicalite obtained from a GCMC simulation [15] using these parameters agreed well with the experimental isotherms obtained by Sun et al. [21] (see Figure 7 in ref. [15]).

Simulations were performed using the DL-POLY classic molecular dynamics simulation package [22]. All simulations were carried out in the NVT ensemble at a temperature of 300 K. The Nose–Hoover thermostat was used to regulate the temperature with a relaxation time of 1 ps. Simulations were run for a total of 2 ns and a calculation time step of 1 fs was used for the simulations. Following the TraPPE-UA convention, a cut-off distance of 14 Å was used. In order to ensure equilibrium of the system, an equilibration time of 0.5 ns was used before the production process. Equilibration of the system was confirmed by inspecting the evolution of total energy and temperature of the system which exhibited stable values within acceptable fluctuation limits after 0.5 ns. During the production process of 1.5 ns, instantaneous positions and velocities of all the atoms/pseudo-atoms were recorded after every 20 fs. 

## 3. Results

### 3.1. Structure

#### 3.1.1. Distribution of the Guest Molecules in a Channel

**Figure 2:**
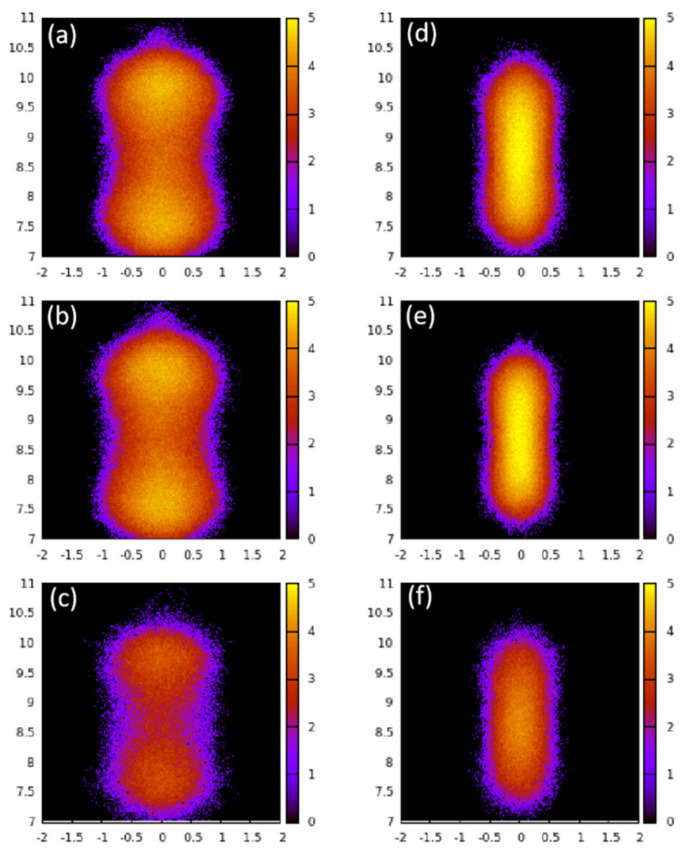
Distribution of guest molecules CO_2_ (**a**–**c**) and ethane (**d**–**f**) in a pore of ZSM-22 at the guest loadings of 72 (**a**,**d**), 52 (**b**,**e**), and 24 (**c**,**f**) molecules in the simulation cell. The intensity represents the logarithm of the number of times a guest molecule is found at a position with the given coordinates.

Figure 2 shows the distribution of the guest molecules in a randomly selected pore of ZSM-22. In this figure, we present the logarithm of the instances when a guest molecule was found at a given location during the entire production time of 1.5 ns. The distribution for CO_2_ is shown on the left, while that for ethane is shown on the right as the loadings increase from bottom to top. Elongated regions of non-zero intensity emulate the elliptically shaped pores that are slightly elongated along the Y-direction (see Figure 1a,c). At the lowest loadings, CO_2_ prefers two locations that are close to the pore wall and roughly symmetrically distributed about the pore axis (two regions of high intensity). As the loading is increased, the separation between these regions becomes somewhat diffused. Ethane molecules on the other hand prefer to reside at the center of the pore (a single high-intensity region located at the center) and exhibit no significant loading dependence of their distribution.

#### 3.1.2. Orientational Structure of the Guest Molecules

**Figure 3:**
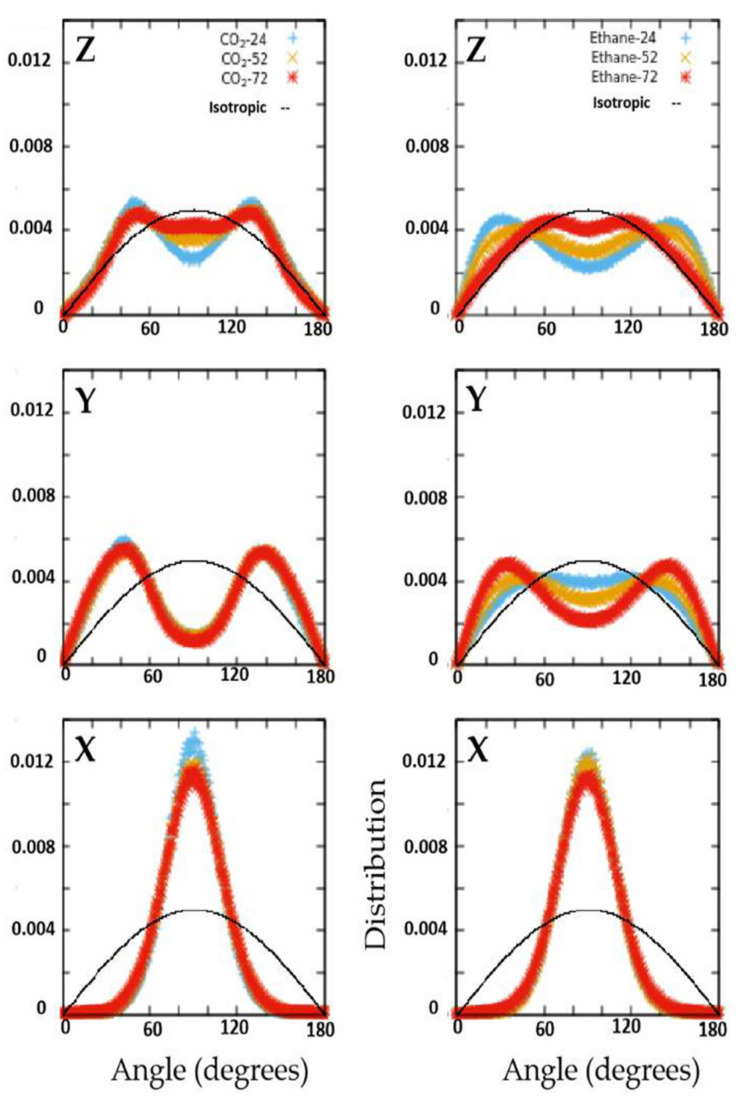
Distribution of angles made by the molecular axis of the guest molecules with respect to Cartesian directions for all loadings investigated in the case of ethane and CO_2_. The distribution for an isotropic case with no preferred orientation is shown as a black curve for reference.

In Figure 3, the orientational distribution of the guest molecules are presented in terms of the angle made by the molecular axis with respect to the different Cartesian directions noted. The plots also show the isotropic distribution expected for a system that exhibits no preferred orientation (black curves). Any deviation from this isotropic distribution indicates orientational ordering where the extent of the deviation is proportional to the degree of orientational ordering. A high degree of orientational order is exhibited by both ethane and CO_2_ adsorbed in the pores of ZSM-22. The order for CO_2_ is approximately independent of loading, whereas higher loading leads to some disorder in the orientational distribution for ethane.

### 3.2. Dynamics

#### 3.2.1. Translational Motion

**Figure 4:**
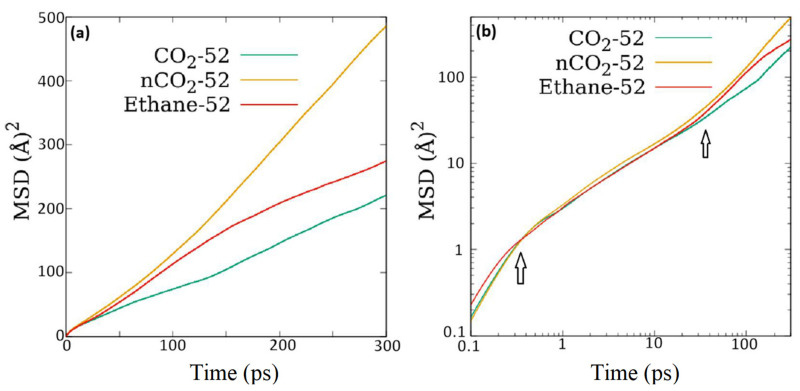
Mean squared displacement (MSD) as a function of time in (**a**) linear scale, and (**b**) log-log scale. Three distinct regions corresponding to ballistic, sub-diffusive, and diffusive motions can be identified in (**b**). nCO_2_ refers to CO_2_ with no electrostatic interactions used in the simulation.

In Figure 4, the mean squared displacement is shown for different guest molecules at the intermediate loading of 52 molecules. While CO_2_ exhibits a lower value of mean squared displacement (MSD) at all times, when the electrostatic interactions are turned off, the resulting MSD values (shown as nCO_2_ curve in the plot) are higher than that for ethane. In the right panel of this figure, the MSD vs. time plot is shown in the log-log plot that helps reveal the behavior of motion as a function of time. This plot exhibits three distinct regions, with separating boundaries marked with arrows. The initial region of rapid increase in MSD lasts for less than 1 ps and represents the ballistic regime of motion. A second region that lasts for a few tens of picosecond shows a relatively slower increase of MSD with time, after which the increase is faster in the third region. The second region represents a restricted sub-diffusion motion, while the third region is the region of diffusive motion. Self-diffusion coefficient D_s_ can be obtained using the Einstein relation by fitting the diffusive region of MSD vs. time curve by a straight line, the slope of which is proportional to D_s_. Values of D_s_ obtained thus for the various systems investigated are reported in Table 3. 

**Figure 5:**
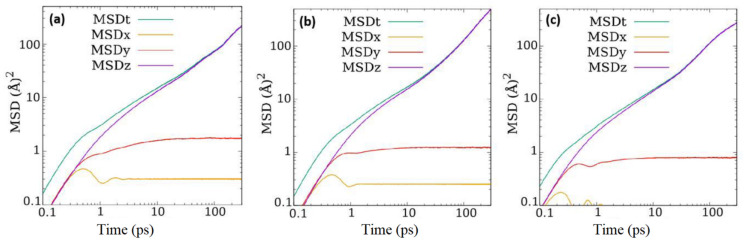
Components of mean squared displacement (MSD) along different directions and the overall MSD (MSDt) for (**a**) CO_2_, (**b**) CO_2_ without electrostatic interactions, and (**c**) ethane in ZSM-22 at a loading of 52 molecules in the simulation cell.

For a nanoporous system with an anisotropic pore network, the diffusivity of the guest molecules can be expected to also exhibit anisotropy. The MSD components calculated along different directions are shown in Figure 5 for the three systems at the intermediate loading of 52 molecules. As expected, the MSD in the directions perpendicular to the direction of the pore-axis is heavily suppressed in all cases. At longer times where the diffusive motion starts, the MSD along the direction of the pore-axis (i.e., Z-direction) is identical to the overall MSD.

#### 3.2.2. Reorientational Motion

**Figure 6:**
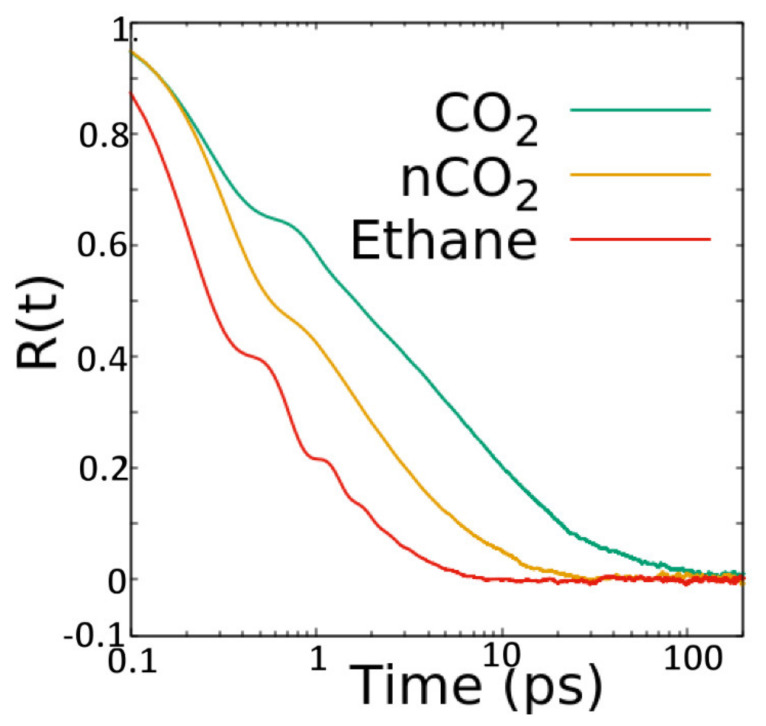
Reorientational correlation function R(t) for CO_2_ and ethane in ZSM-22 at a loading of 52 molecules in the simulation cell. In the legend, nCO_2_ indicates results from the simulation of CO_2_ without electrostatic interactions.

Rotational or reorientational motion of the guest molecules was investigated by following the orientation of the molecular axis over time. In particular, we calculated the correlation function R(t) = <***u***(0).***u***(t)> of a unit vector ***u*** attached to the molecular axis. The R(t) for the representative systems investigated is shown in Figure 6. The R(t) vs. time curves can be divided into two distinct regions separated by a wobble at around 1 ps. The second region represents the long-time overall rotation of the guest molecules. To obtain the time scales of rotational motion, we fitted the second region of R(t) with an exponential function R(t) = *a*exp(−t/τ) + *c* with *a*, τ, and *c* as fitting parameters. The time constants τ obtained thus are listed in Table 4.

### 3.3. Effect of Connecting the Pores Artificially by Inserting Inter-Crystalline Space

**Figure 7:**
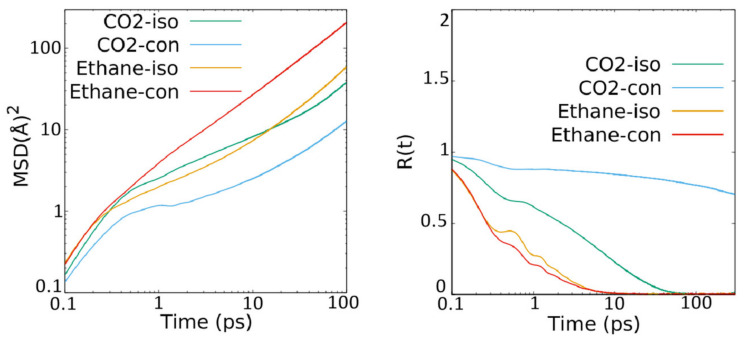
MSD (**left**) and R(t) (**right**) of ethane and CO_2_ in ZSM-22 at a loading of 72 molecules in the simulation cell. In the legend, the suffices “iso” and “con” are used to distinguish between the simulations involving unmodified ZSM-22 with isolated pores and ZSM-22 modified by inserting inter-crystalline space that results in the pores being connected via the inter-crystalline space.

ZSM-22 has parallel channel-like pores running in the Z-direction that are isolated from each other. The effects of inter-connecting these pores was studied by inserting extra empty space in the simulation on top and bottom of the simulation cell. These empty spaces provide a transit for the molecules in one pore to migrate to another pore. The effect of this inter-connection on the MSD and R(t) of both CO_2_ and ethane are shown in Figure 7 (left and right panels respectively). The effect of this pore connectivity via inter-crystalline spacing is different for the two guest molecules. While both translational and rotational motion of CO_2_ are suppressed on connecting the pores, ethane represents an enhancement of translational motion but a suppression of rotational motion when the pores are connected via the inter-crystalline space. The last of these effects, i.e., suppression of rotational motion of ethane in connected pores is evident in Figure 7 (right panel) that exhibits a slower decay in the second region of R(t) for ethane in the connected pores as compared to that for isolated pores.

## 4. Discussion

The structural and dynamical behavior of both fluids mimic the structure of the pores. As seen in Figure 2, the fluid molecules tend to occupy the pores in positions that are elongated along the Y axis, in consistency with the pores that are slightly elongated along the *Y* axis (see Figure 1). This slight elongation along the *Y* axis also gives rise to preference to orient perpendicular to the *X*-axis (see Figure 3). While CO_2_ molecules prefer to lie making an angle of roughly 45 degrees with respect to both Y- and Z-directions, the ethane molecules show a preference to make smaller (or larger) angles with Z (or Y-directions) at lower loadings, progressively shifting towards larger (or smaller) angles with Z (or Y-directions). Thus, while ethane exhibits a range of orientations with respect to the pore axis (and hence pore wall) depending on the loading, CO_2_ molecules show a preference to lie making an angle of 45 degrees with the pore surface irrespective of the loading. A preference for this orientation with respect to the substrate has been observed for CO_2_ in several other systems [15,23]. 

The results presented here show that the electrostatic interactions play an important role in the behavior of CO_2_ under confinement. This has been reported earlier in several other studies [15,24]. The quadrupole moment of CO_2_ is responsible for selective adsorption of CO_2_ on silica surfaces, leading to reduced mobility. This restriction on the motion of CO_2_ due to the electrostatic interactions is relatively weaker as compared to the restriction imposed on slightly larger polar molecules like acetonitrile and acetaldehyde [6] that are rendered practically immobile in the channels of ZSM-5 of similar dimensions as in ZSM-22 investigated here. 

A quick comparison of the overall self-diffusion coefficients reported in Table 3 here with those for the two fluids reported in ZSM-5 [14] suggests that although the diffusivity of ethane in ZSM-22 is same as that in ZSM-5, that of CO_2_ is slightly reduced in ZSM-22 compared to that in ZSM-5. However, there are some important caveats to consider. As stated in the introduction, the pore network of ZSM-22 is similar in dimensions to that in ZSM-5 but without inter-connections between different channels. The straight channel-like pores in ZSM-22 are aligned along the crystallographic axis *c* and are analogous to the straight channels in ZSM-5 that run parallel to *b* axis. One direct consequence of the difference in the pore structure in ZSM-22 and ZSM-5 is that while in the former, the mean squared displacement at longer time is entirely along the direction of straight channels, that in the latter is distributed along all directions even though the component along the direction of straight channels is dominant. This is because of the existence of sinusoidal channels that run perpendicular to the straight channels in ZSM-5, giving rise to motion along the plane perpendicular to the straight channels. These sinusoidal channels also provide a connection between the straight channels. The effect of the presence of this connection between the pores in ZSM-5 or their absence in ZSM-22 can be understood by comparing the one-dimensional diffusivity of fluid molecules along the pore direction, i.e., in the Y-direction in ZSM-5 and in Z-direction in ZSM-22. For CO_2_ loaded in ZSM-5 at partial gas pressures between 0.1 to 100 bar, this diffusivity was in the range of 66 to 12 × 10^−10^ m^2^/s, while for ethane in ZSM-5, it was in the range 71 to 5.5 × 10^−10^ m^2^/s for these partial pressures [14]. The different loadings as reported here also correspond roughly to the same range of partial pressures. As the MSD along the pore axis in ZSM-22 is identical to the overall MSD at long times, the overall self-diffusion coefficients listed in Table 3 can be used as a measure of the diffusion coefficient along the pore axis. However, it should be noted that the self-diffusion coefficients along the pore axis calculated for ZSM-5 as mentioned above were obtained by considering one-dimensional motion while those reported in Table 3 for ZSM-22 are obtained considering 3-D motion. Thus, a fair comparison between the two sets of diffusion coefficients (those for ZSM-5 and ZSM-22) requires multiplying the values listed in Table 3 by a factor of 3 (The self-diffusion coefficient is obtained via Einstein relation as D_s_ = MSD/(2n_d_t), where n_d_ is the number of dimensions; n_d_ = 3 for the overall D_s_ as listed in Table 3; n_d_ = 1 for one-dimensional diffusion. Thus, to convert the 3-D D_s_ values listed in Table 3 to a 1-D self-diffusion coefficient, the former needs to be multiplied by 3). With this normalization, we obtain the one-dimensional self-diffusion coefficients of CO_2_ and ethane in the direction of the pore axis of ZSM-22 in the range of 97 to 15.6 × 10^−10^ and 162 to 16.6 × 10^−10^ m^2^/s, respectively. These self-diffusion coefficients along the pore axis in ZSM-22 are thus higher than those in ZSM-5, suggesting that pore connectivity hinders the diffusivity. This could be because of the collisions with the cross-running molecules in the sinusoidal channels that slow down the motion of molecules in the straight channels of ZSM-5, while in absence of pore connectivity, the molecules in the channel-like pores of ZSM-22 encounter no such collisions and hence mov without any hindrance. It has been found that CO_2_ molecules show a preference for partitioning in the intersections of the straight and sinusoidal channels in ZSM-5, whereas ethane molecules are more likely to be found in the sinusoidal channels at high loadings [14]. However, in another study [25] it was found that the residence auto-correlation function of ethane in the intersections of ZSM-5 decayed slower than those for ethane in straight and sinusoidal channels by a factor of more than 2. This means that even though the energetics favor the partitioning of ethane in sinusoidal channels, once they reach the intersections, they are likely to spend a significant amount of time there, probably undergoing a rattling motion. This rattling motion of the guest molecules in the intersection provides a hindrance to the motion of other guest molecules through the straight channels. 

While the translational motion of both ethane and CO_2_ is facilitated by the absence of pore connectivity, the effect of pore connectivity on the rotational motion is different on the two fluids. For ethane, the time scales of rotational motion as listed in Table 4 (between 1.85 and 1.89 ps) are smaller than those for ethane in ZSM-5 (between ~2 and 12 ps) [14], suggesting a faster rotation for ethane in ZSM-22. In contrast to this, the rotational time scales for CO_2_ in ZSM-22 (between 13 and 42 ps) are larger than those in ZSM-5 (between 7 and 22 ps) [14], suggesting a slower rotation of CO_2_ in ZSM-22. Rotational motion of both guest molecules in ZSM-22 is enhanced at higher loadings. A similar enhancement of rotational motion at higher loading was also observed for ethane and CO_2_ in ZSM-5 [14,25] and was explained in terms of a decreased orientational order at higher loadings.

In the absence of inter-connections between the pores of ZSM-22, we added artificial inter-connections by exposing the pores to common inter-crystalline space. The artificial addition of such inter-crystalline space has been earlier used to emulate the powder samples in simulations [15,26,27,28]. In the present case, this inter-crystalline space effectively provided a connection between the straight channel-like pores of ZSM-22. Comparing the values of overall self-diffusion coefficients in ZSM-22 with and without pores connected via inter-crystalline space listed in Table 3, it can be seen that the effects of this artificially imposed pore connectivity is different on the two fluids. While ethane diffusion is facilitated by pore connectivity, that of CO_2_ is suppressed significantly. However, we note that as the inter-crystalline space added for connecting the pores provides a three-dimensional slab-like space in which the molecules are free to move in X- and Y-directions in addition to the Z-direction, a fair comparison with the case of isolated pores in ZSM-22 where motion occurs only along the Z-direction would require limiting the comparison to diffusivity along the Z-direction. Self-diffusion coefficients of ethane and CO_2_ along the Z-direction obtained for ZSM-22 modified with inter-crystalline space are, respectively, 19.92 × 10^−10^ and 2.01 × 10^−10^ m^2^/s. Compared to the values of 16.65 × 10^−10^ and 15.54 × 10^−10^ m^2^/s for the self-diffusion coefficient along Z-direction of ethane and CO_2_ in ZSM-22 with isolated pores, (obtained by normalizing the values listed in Table 3 for one-dimensional motion), we see that the effect of pore connectivity via inter-crystalline space is indeed different for the two fluids—enhancement of diffusivity for ethane and its suppression for CO_2_ in ZSM-22. Unlike the difference in the effects of pore connectivity via inter-crystalline space on translational motion of ethane and CO_2_, the rotational motion of both ethane and CO_2_ were found to be suppressed by inter-crystalline space (Table 4).

**Figure 8:**
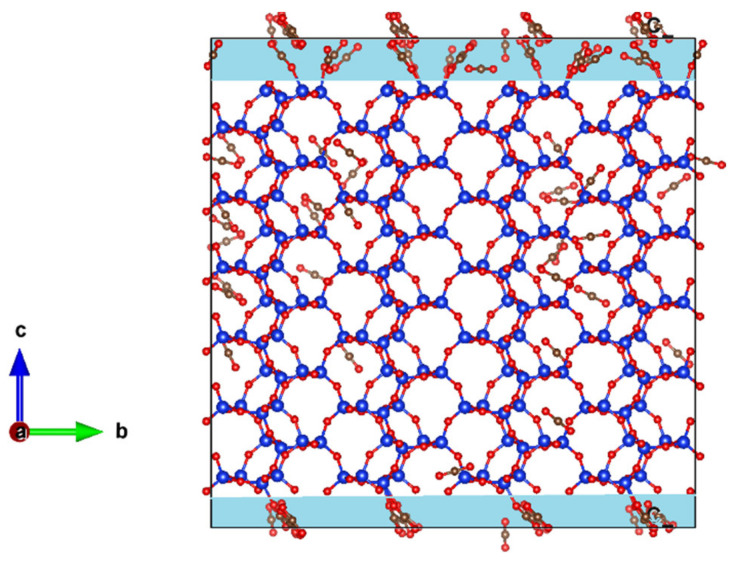
Snapshots in X–Z plane from the simulation of CO_2_ in ZSM-22 with empty space inserted in the Z-direction. Blue, red, and gray spheres respectively represent Si, O, and C atoms. More CO_2_ molecules can be seen strongly adsorbed on the surface of ZSM-22 in the inter-crystalline space (shown in light blue) as compared to inside the pores.

In Figure 8 we show a snapshot of CO_2_ adsorbed in ZSM-22 with inter-crystalline space inserted at the bottom and top of the simulation cell along the Z-direction. The corresponding snapshot for ethane in this system is shown in Figure 1d. A clear distinction can be seen between the behavior of ethane and CO_2_ in this system by comparing Figure 8 and Figure 1d. While ethane molecules prefer to be adsorbed in the ZSM-22 pores, more CO_2_ molecules can be seen adsorbed on the surface of the crystallite in the inter-crystalline space, suggesting a preference for the surface adsorption compared with adsorption in the crystalline pores. This is consistent with the difference observed between ethane and CO_2_ adsorption in the inter-crystalline space of ZSM-5 [15]. The curvature of the silica surface plays an important role in the preferential adsorption of CO_2_ on the surface vis-a-vis the pores. While the silica surface in the vicinity of the inter-crystalline space is flat, that in the pores has a strong curvature such that a CO_2_ molecule is wrapped around by it. As a result, the silica atoms on the opposite sides compete for and nullify their interaction with CO_2_, thereby freeing the CO_2_ molecule to move along the pore axis. This nullification of strong surface interactions by opposite pore surfaces has been found to enhance the diffusivity of guest molecules in zeolite pores and is termed as the “levitation effect” [29]. Strong adsorption of CO_2_ on the crystallite surface has important consequences for the dynamical behavior. Connecting the pores of ZSM-22 by inserting inter-crystalline space leads to a suppression of both translational as well as rotational dynamics of CO_2_, while with weaker substrate–fluid interactions in the case of ethane, the inserted space provides greater freedom to ethane to move between the pores, thus enhancing its mobility. Further, the intersection between the channels and the interconnecting slab-like space is open and the guest molecules show no preference to localize at these intersections, in contrast to the case of ZSM-5. This reduces the probability of collisions considerably and thereby in absence of collisions, the guest molecules in the straight channels of ZSM-22 are free to move in absence of any hindrance. This suggests that the effects of interconnecting the pores depend upon the way the pores are connected.

## 5. Conclusions

MD simulations of ethane and CO_2_ reported here, in combination with a previous study involving ZSM-5, help us understand the role of pore connectivity in determining the structural and dynamical behavior of the confined fluids. We found that the effects of pore connectivity depend not only on the type of the fluid under confinement but also the way the pore connectivity is imposed. While the translational motion of both ethane and CO_2_ in ZSM-22 is suppressed as a result of connecting the pores by perpendicular quasi one-dimensional pores of similar dimensions, the effect of connecting the pores by inserting a slab-like 2-dimensional inter-crystalline space is different on the translational motion of the two fluids. For ethane, pores connected via inter-crystalline space facilitate translational motion but suppress rotational motion, whereas in the case of CO_2_, both types of motion are suppressed by pore connection due to the strong interaction of CO_2_ with the surface of the substrate that reduces its mobility. This computational study is a first step toward understanding the interplay of fluid–substrate interactions, pore connectivity, and pore dimensionality. The effects of pore connectivity involve a variety of other factors including the degrees of connectivity, pore volume, and surface areas, which are not addressed in this work but will be addressed in future works.

## Figures and Tables

**Table 1:** Lennard–Jones parameters for the CO_2_–ZSM-22 interactions.

Interacting Molecules LJ Parameters	Si-C	Si-O_C_	O-C	O-O_C_	C-C	C-O_C_	O_C_-O_C_
*ε_ij_* KJ/mol	0.00131	0.00225	0.38163	0.65359	0.224	0.38362	0.657
*σ_ij_* Å	3.051	3.176	2.983	3.108	2.8	2.925	3.05

**Table 2:** Lennard–Jones parameters for ethane–ZSM-22 interactions.

Interacting Molecules LJ Parameters	Si-CH_3_	O-CH_3_	CH_3_-CH_3_
*ε_ij_* KJ/mol	0.00251	0.72795	0.815
*σ_ij_* Å	3.5264	3.458	3.750

**Table 3:** Self-diffusion coefficients in the units of 10^−10^ m^2^/s obtained from the long-time behavior of MSD from different simulations.

System	72 Molecules	52 Molecules	24 Molecules
Ethane in ZSM-22	5.55 ± 0.01	17.47 ± 0.01	54.06 ± 0.02
CO_2_ in ZSM-22	5.18 ± 0.01	11.43 ± 0.01	32.61 ± 0.52
Chargeless CO_2_ in ZSM-22	-	30.14 ± 0.01	-
Ethane in ZSM-22 with pore connecting space	33.91 ± 0.01	-	-
CO_2_ in ZSM-22 with pore connecting space	1.56 ± 0.01	-	-

**Table 4:** Time constants τ (in ps) of the decay of the rotational correlation functions above 1 ps.

System	72 Molecules	52 Molecules	24 Molecules
Ethane in ZSM-22	1.85 ± 0.01	1.76 ± 0.01	1.89 ± 0.02
CO_2_ in ZSM-22	13.3 ± 0.02	24.71 ± 0.11	42.2 ± 0.24
Chargeless CO_2_ in ZSM-22	-	2.87 ± 0.02	-
Ethane in ZSM-22 with pore connecting space	2.16 ± 0.01	-	-
CO_2_ in ZSM-22 with pore connecting space	46.13 ± 0.01	-	-

## Data Availability

The authors confirm that the data supporting the findings of this study are available within the article.
